# Patients’ Awareness of the Ocular Side Effects of Isotretinoin Therapy: A Study From Saudi Arabia

**DOI:** 10.7759/cureus.24628

**Published:** 2022-04-30

**Authors:** Razan M AlMasoudi, Reem K Bahaj, Amal A Kokandi

**Affiliations:** 1 Medicine and Surgery, Ibn-Sina National College, Jeddah, SAU; 2 Medical Laboratory Technology, Faculty of Applied Medical Sciences, King Abdulaziz University, Jeddah, SAU; 3 Family Medicine, Faculty of Medicine, King Abdulaziz University, Jeddah, SAU; 4 Dermatology, King Abdulaziz University Hospital, Jeddah, SAU

**Keywords:** ophthalmic side effects, awareness, practice, ocular manifestations, : isotretinoin

## Abstract

Introduction

Isotretinoin is one of the most commonly prescribed drugs among dermatologists because it's used in the treatment of Acne vulgaris. Despite having an adequate safety profile, isotretinoin-related adverse events are common, with ocular manifestations being one of them. Although being generally mild, ophthalmologic manifestations associated with isotretinoin may cause significant ocular morbidity.

Objectives

The objective is to evaluate the awareness of the ocular side effects of isotretinoin treatment in patients with acne and to understand the treating physicians' practice of prescribing isotretinoin from the patient's point of view.

Method

A descriptive cross-sectional study was conducted on a Saudi Arabian population from June to September 2021. We used a self-administered questionnaire to collect data on drug dose, treatment duration, ocular adverse effects, patients' awareness, and patients' perception of treating physicians' practice.

Results

Of 1,157 responders, 452 (39.1%) consisted of participants who met the inclusion criteria, therefore they were enrolled in the study. Out of our responders, 308 (68.1%) were women. Approximately, 91.6% of participants had a dermatologist's prescription for isotretinoin. Eye dryness was the most commonly reported ocular adverse effect (83.4%). Blepharitis and conjunctivitis were the most commonly diagnosed complications after starting isotretinoin (5.4% for each).

Approximately, 71.2% participants were not advised to see an ophthalmologist for ophthalmological screening. Physicians asked only 78 (17.3%) respondents about a recent refractive surgery before starting isotretinoin or if they intended to undergo a refractive surgery in the next six months. Approximately, 57.5% participants were unaware of possible contact lens intolerance. Lubricant eye drops were prescribed to 305 (67.5%) respondents during isotretinoin treatment. Approximately, 36.7% participants were advised to consult their treating physicians if they had any serious ocular signs or symptoms.

Approximately, 67.9% participants reported their awareness of the ocular side effects of isotretinoin therapy. However, 236 (52.2%) participants responded that they were not provided with sufficient information on the ocular side effects of isotretinoin therapy.

## Introduction

Acne vulgaris is a common skin condition affecting 9.4% of the global population. It has a high incidence among young adults and adolescents. It may cause facial scarring often resulting in emotional distress [1,2]. Although mild to moderate acne vulgaris can be treated with several topicals or oral antibiotics, a severe form of the disease can be challenging to treat with these approaches. Oral isotretinoin is considered a first-line treatment for severe forms of acne vulgaris [3].

Isotretinoin has proven to be an effective treatment for acne vulgaris with an overall safety profile. However, it may cause adverse effects, including ocular side effects, such as dry eye disease (DED), blepharitis, and conjunctivitis [4].

Blepharoconjunctivitis is the most common isotretinoin-induced side effect, occurring in 20%-50% of patients. There are reports of other dangerous side effects, including retinal dysfunction and papilledema [5,6].

Although isotretinoin therapy may have significant side effects, multiple studies have shown a lack of awareness of these side effects. A study conducted in the western region of Saudi Arabia revealed that 15%-50% of patients who used oral isotretinoin for acne lacked knowledge about the side effects of isotretinoin treatment [7].

Another study in Al Madina, Saudi Arabia, showed that 45.6% of participants were not notified about the side effects of the drug. Additionally, the study showed that the major sources of information on isotretinoin were friends (45.2%) and social media (41.8%) [8].

Laser-assisted in situ keratomileusis (LASIK) is an ophthalmologic corneal procedure and an effective surgical treatment of refractive errors [9]. However, dry eye is a well-known side effect of LASIK; up to 95% of patients suffer from dry eyes following the procedure [10]. It was shown that Isotretinoin can induce dry eye, and there's a relative contraindication in using it after LASIK surgery [11,12]. Some studies have shown the opposite. The use of Isotretinoin can be safe post dermatosurgical and laser interventions and the contraindication might need revision [13,14]. 

Unfortunately, dermatologists who prescribe isotretinoin are still not well aware about the ocular side effects of the drug. Previous studies have emphasized the need for routine screening for recent LASIK operation and indicated that patients should wait six months after undergoing a refractive eye surgery to allow for corneal healing before starting Isotretinoin [15].

According to a survey among British dermatologists, 89% were unaware of possible isotretinoin-induced eye complications in patients who had undergone LASIK surgery within the previous six months [16]. As stated in another study in Saudi Arabia, 44% of dermatologists failed to notify their patients that they should avoid laser refractive surgery for six months after discontinuing isotretinoin. In addition, the majority of dermatologists stated that they never sent their patients to an ophthalmologist if lubricating drops were administered [17]. We lack data on the current awareness of the ocular side effects of oral isotretinoin among patients with acne vulgaris.

The aim of this study was to evaluate the awareness of the ocular side effects of isotretinoin treatment in patients with acne and to understand the treating physicians' practice of prescribing isotretinoin from the patient's point of view.

## Materials and methods

A cross-sectional study was conducted among patients who used isotretinoin for treating acne in different regions of Saudi Arabia from June to September 2021. The Institutional Review Board of King Abdulaziz University (approval number 351-21) authorized this study. We obtained informed consent from all participants.

A structured questionnaire was administered to collect data to assess the awareness of the ocular side effects of isotretinoin treatment in patients with acne who received isotretinoin in the past or are currently on isotretinoin for more than four months. and to understand the treating physicians' practice of prescribing isotretinoin from the patient's point of view.

Exclusion criteria

The criteria excluded less than four months of isotretinoin treatment, less than 18 years of age, and an ocular disease before starting isotretinoin treatment.

Previously, a pilot study was initially conducted among 10 medical students to measure the face validity of the questionnaire. Additionally, an expert dermatologist reviewed the questionnaire. We distributed the questionnaire throughout different social media networking sites, such as WhatsApp, Telegram, Twitter, and Snapchat, taking the advantage of their simplicity of use and accessibility. Information obtained with the questionnaire includes socio-demographic characteristics; the prescription, including dose and treatment duration; any ocular side effects or complications; notification of ocular side effects or complications; patients' awareness; and patients' perspective on treating physicians' practice.

Descriptive statistics were used, such as frequency distribution, percentage, mean, and standard deviation, to analyze the collected data. We used the chi-square test to examine the relationship between isotretinoin awareness and age, gender, and education, as well as the relationship between the prescribed dose and side effects/complications experienced. A p-value of 0.05 was considered statistically significant. We performed all data analyses using Statistical Packages for Software Sciences version 24 (IBM Corp, Chicago, IL).

## Results

Out of 1,157 respondents, 452 (39.1%) who met the inclusion criteria were included in the study.. Those who had never used isotretinoin before constituted 285 (24.6%), participants who had an ocular disease 135 (15.5%), and participants who used isotretinoin for less than four months 255 (34.6%) and were therefore excluded from the study. 

Most of the included respondents were women 68.1%, and the rest were men. The majority of participants were aged between 18 and 25 years (65.7%). We observed that 91.6% of participants had a dermatologist's prescription for isotretinoin, whereas 3.3% of responders took isotretinoin by themselves and 5.1% took it based on a non-dermatologist physician's prescription. The most frequently administered isotretinoin dose was 20-30 mg in 38.3% of responders, followed by 30-40 mg in 36.5% of responders (Table 1).

**Table 1:** Respondents' demographic characteristics and isotretinoin-related questions

Demographic characteristic		No. (%)	
		Count	%
Gender	Female	308	68.1
	Male	144	31.9
Age group	18-25 years old	297	65.7
	26-35 years old	120	26.5
	36-45 years old	23	5.1
	Above 45 years old	12	2.7
Dose (mg)	<10	29	6.4
	10-20	173	38.3
	30-40	165	36.5
	50-60	20	4.4
	70 or more	4	0.9
	Unspecified	61	13.5
Isotretinoin prescribed by	Dermatologist	414	91.6
	Another doctor (not dermatologist)	23	5.1
	Self-prescription	15	3.3

**Figure 1:**
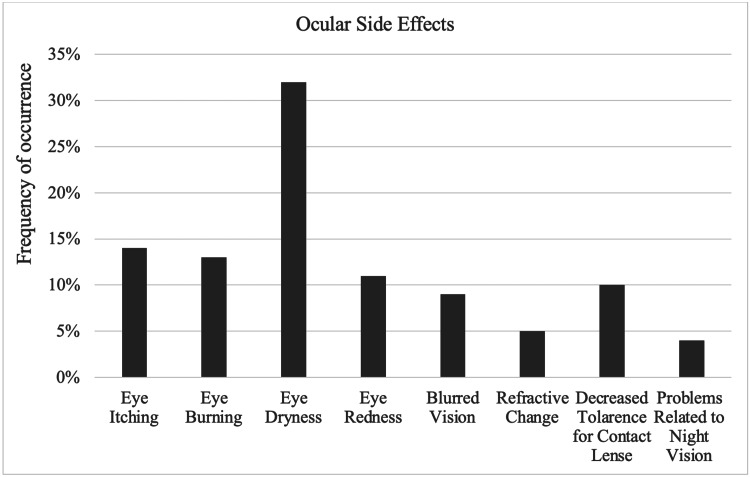
Ocular side effects experienced by respondents

The questionnaire had numerous options for ocular adverse effects. Eye dryness was the most commonly reported side effect 83.4%. On the other hand, problems related to color vision and night vision were the least common side effects 11.7% (Figure 1).

**Figure 2:**
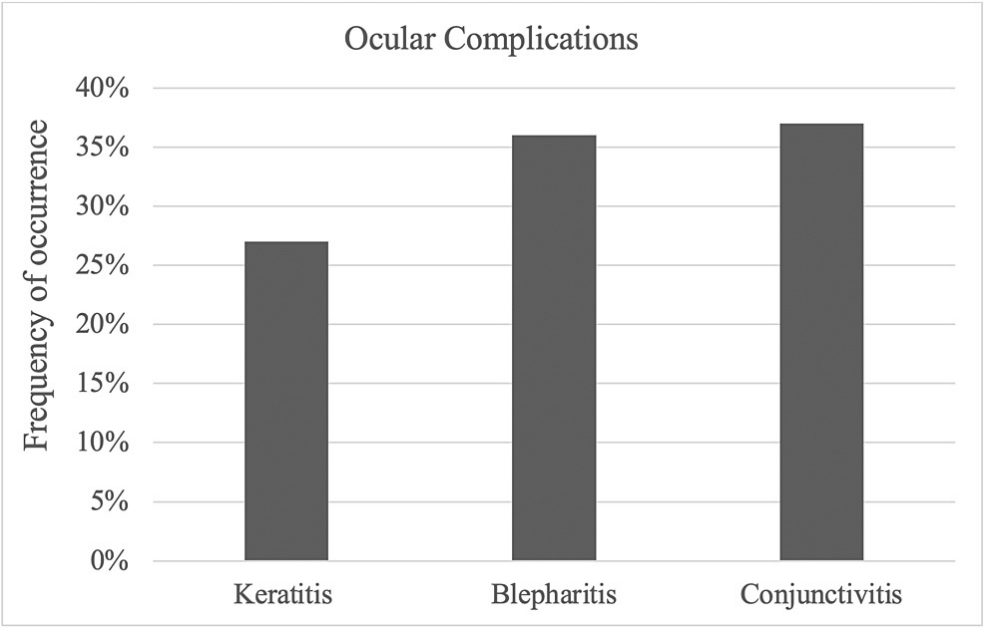
Ocular complications experienced by respondents

Moreover, several respondents reported eye pain and eye swelling when asked about any other side effects they experienced while using isotretinoin. Blepharitis and conjunctivitis (5.4% for each) were the most commonly diagnosed complications after starting isotretinoin (Figure 2).

Physicians asked only 17.3% respondents if they had undergone a vision correction operation in the previous six months or planned to undergo one in the next six months. Approximately 71.2% respondents were not advised to see an ophthalmologist for ophthalmological assessment by their treating physicians. In addition, 57.5% participants were unaware of possible contact lens intolerance, almost 67.5% participants were prescribed a lubricating eye drop. Furthermore, 36.7% were advised to consult their treating physicians if they had any serious ocular indications or symptoms (Table 2).

**Table 2:** Treating physician practice

Dermatologist’s practice		No. (%)	
		Count	%
Did your treating physician suggest you to visit an ophthalmologist for an eye examination?	Yes	83	18.4
	No	322	71.2
	I don’t remember	47	10.4
Were you asked if you had undergone a vision correction operation in the past 6 months or intended to undergo one within the next 6 months?	Yes	78	17.3
	No	305	67.5
	I don’t remember	69	15.3
Were you informed about possible contact lens intolerance?	Yes	125	27.7
	No	260	57.5
	I don’t remember	67	14.8
Did your treating physician prescribe a lubricant eye drop for you?	Yes	305	67.5
	No	116	25.7
	I don’t remember	31	6.9
Did your treating physician suggest that you should see him as soon as possible in case of any significant ocular signs or symptoms?	Yes	166	36.7
	No	206	45.6
	I don’t remember	80	17.7

Around 67.9% participants reported that they were aware of the adverse ocular effects of isotretinoin treatment although 52.2% participants stated that they were not provided with enough information on the ocular side effects of the drug.

Finally, this study showed that women had better awareness of different ocular side effects compared to men (68.1% vs. 31.9%, p=0.043), indicating a significant relationship between gender and the total awareness of the ocular side effects of isotretinoin treatment.

## Discussion

Since 1982, isotretinoin (13-cis retinoic acid) has been used to treat severe or persistent acne. Although isotretinoin is an effective treatment for persistent acne, it has several adverse effects. These ocular side effects include DED, blepharitis, and conjunctivitis [4-6]. It is important for physicians to be mindful of the adverse effects of the drug [18].

In this study, we assessed patients' awareness of the ocular side effects of isotretinoin treatment and physicians' practice of prescribing isotretinoin. Out of 452 participants included in this study, approximately 91.6% had a dermatologist's prescription for isotretinoin. The possession of an isotretinoin prescription was expected, given that a prescription is required for a pharmacy to dispense the drug. This finding is comparable to those of other studies in Saudi Arabia [8,19].

The majority of participants chose eye dryness as the ocular side effect of isotretinoin treatment 83.4%. These findings are consistent with previously published data on the outcomes of LASIK and photorefractive keratectomy in patients taking isotretinoin [20].

We also found that blepharitis and conjunctivitis (5.4% for each) were the most common complications diagnosed by an ophthalmologist after starting isotretinoin treatment. These findings were also consistent with a study that investigated 1,741 cases of ocular side effects associated with isotretinoin usage. The most common ocular complaint was blepharoconjunctivitis [21].

When assessing treating physicians' practice, we found that treating physicians did not advise 71.2% of respondents to see an ophthalmologist for an eye examination. In addition, 57.5% of participants were unaware of possible contact lens intolerance. Results from previous studies assessing dermatologists' practice regarding the use of isotretinoin found that the majority of dermatologists did not consider it necessary to refer their patients to an ophthalmologist for detecting and treating DED [17].

DED is a well-known side effect of LASIK. Since isotretinoin can induce eye dryness, undergoing the procedure while on isotretinoin is contraindicated [11,12]. Unfortunately, physicians asked only 17.3% of respondents if they had undergone a vision correction operation in the previous six months or planned to undergo one in the next six months.

Around 67.9% of the participants showed good knowledge regarding the ocular side effects of isotretinoin treatment. However, when asked about their major source of information, only half of these respondents mentioned their treating physicians as their source of information, while the other half received information on isotretinoin from internet or social media, friends or relatives, and brochure attached with the medication.

The primary limitation of this study is that the data were collected online via a self-reported survey which might be influenced by reporting bias. Also, medical terms and diagnoses used in the survey might not reflect the true condition of the participants due to lack of cross-reference with their medical reports from their respective ophthalmologists. Furthermore, patients were managed by different dermatologists. Since the data entirely depend on patients’ honesty, the study has limited generalizability.

## Conclusions

In conclusion, even though our population was generally aware of the ocular side effects of isotretinoin use, more than half were not aware of possible contact lens intolerance. And less than a third were asked by a dermatologist about recent refractive surgery before beginning the course or if they planned to have refractive surgery six months after the course ended. This shows that dermatologists have low awareness about the proper refractive surgery practices when prescribing isotretinoin. Improving dermatologists' understanding of isotretinoin prescription practice is critical for preventing ocular damage. We suggest that there is a need to have any patient being considered for treatment with isotretinoin be reviewed by an ophthalmologist before the commencement of treatment, during, and after treatment.
